# Association of vitamin B_12_ deficiency in a dementia cohort with hippocampal atrophy on MRI

**DOI:** 10.1016/j.tjpad.2025.100265

**Published:** 2025-07-17

**Authors:** Asako Ueno, Tadanori Hamano, Miwako Nagata, Tomohisa Yamaguchi, Yoshinori Endo, Soichi Enomoto, Hirohiko Kimura, Masamichi Ikawa, Osamu Yamamura, Daiki Yamanaka, Yohei Kimura, Yasunari Nakamoto, Yasuhiro Nishiyama

**Affiliations:** a Department of Neurology, Faculty of Medical Sciences, University of Fukui, 23-3 Matsuokashimoaizuki, Eiheiji-cho, Yoshida-gun, Fukui 910-1193, Japan; b Department of Neurology, Fukui Saiseikai Hospital, 7-1 Funabashi, Wadanaka-cho, Fukui-city, Fukui 918-8503, Japan; c Department of Gerontology, Faculty of Medical Sciences, Kanazawa Medical University, 1-1 Daigaku, Uchinada, Kahoku, Ishikawa 920-0293, Japan; d Life Science Innovation Center, Faculty of Medical Sciences, University of Fukui, 23-3 Matsuokashimoaizuki, Eiheiji-cho, Yoshida-gun, Fukui 910-1193, Japan; e Department of Neurology, Nakamura Hospital, 4-28 Tenno-cho, Echizen-city, Fukui 915-0068, Japan; f Department of Radiology, Faculty of Medical Sciences, University of Fukui, 23-3 Matsuokashimoaizuki, Eiheiji-cho, Fukui 910-1193, Japan; g Department of Community Health Science, Faculty of Medical Sciences, University of Fukui, 23-3 Matsuokashimoaizuki, Eiheiji-cho, Yoshida-gun, Fukui 910-1193, Japan; h Department of Community Medicine, Faculty of Medical Science, University of Fukui, 23-3 Matsuokashimoaizuki, Eiheiji-cho, Yoshida-gun, Fukui 910-1193, Japan; i Kimura Hospital, 57-25 Kitakanazu, Awara-city, Fukui 919-0634, Japan; j Second Department of Internal Medicine, Faculty of Medical Sciences, University of Fukui, 23-3 Matsuokashimoaizuki, Eiheiji-cho, Yoshida-gun, Fukui 910-1193, Japan

**Keywords:** Vitamin B_12_, MMSE, Hippocampal atrophy, MRI-VSRAD, Z-score

## Abstract

**Background:**

Vitamin deficiencies have been reported to cause brain atrophy. Hippocampal atrophy has been well reported in patients with dementia including Alzheimer’s disease.

**Objectives:**

To investigate the association between hippocampal atrophy and vitamin deficiency

**Design:**

Cross sectional study

**Setting:**

Three sites in one country

**Participants:**

Overall, 567 patients who visited an outpatient dementia clinic and underwent MRI-VSRAD (Voxel-Based Specific RegionalAnalysis System for Alzheimer's Disease) were included in this study.

**Intervention:**

Patients with a hippocampal atrophy Z-score of < 2 were classified as normal (*n* = 323), and those with a Z-score of ≥ 2 were diagnosed with hippocampal atrophy (*n* = 244).

**Measurements:**

Vitamin B_12_, folic acid, vitamin B_1_, homocysteine, HbA1c, and creatinine levels were measured and their association with hippocampal atrophy was assessed. Age, MMSE (Mini Mental State Examination), and hippocampal atrophy were also evaluated.

**Results:**

In the hippocampal atrophy group, the frequency of vitamin B_12_ deficiency was higher (*p* < 0.022), MMSE score was lower (*p* < 0.0001), and age was higher (*p* < 0.0001) than that in the normal group (Mann-Whitney *U test*). Patients with vitamin B_12_ deficiency (odds ratio, 3.46) and low MMSE score (odds ratio, 2.24) had an increased risk of hippocampal atrophy.

**Conclusion:**

Vitamin B_12_ deficiency was associated with hippocampal atrophy detected by VSRAD analysis. Therefore, early vitamin B_12_ supplementation should be considered in patients with deficiencies to reduce dementia risk.

## Highlights

• This analysis consists of 567 participants revealed that vitamin B12 deficiency, not folate or vitamin B1 deficiency was associated with hippocampal atrophy detected by MRI-VSRAD analysis.
• Vitamin B12 deficiency was associated with hyperhomocysteinemia.
• Clear correlations were observed between hippocampal atrophy and vitamin B12 value.

## 1 Introduction

Deficiencies of vitamins including vitamin B_12_, folate, and vitamin B_1_ have been proposed to cause treatable dementia [1,2]. B vitamins, which are part of the metabolic network, are involved in nutrient signaling and biosynthesis, oxidation–reduction homeostasis, and epigenetics. These vitamins play an essential role in the regulation of cell proliferation, stress resistance, and embryogenesis. Additionally, vitamin B_12_ deficiency is common in the older population, and may affect neural function through impaired myelination [3].

Homocysteine is converted to methionine by methionine synthase. Vitamin B_12_ is used by methionine synthase as a cofactor. Vitamin B_12_ shortage can lead to hyperhomocysteinemia [4]. Folic acid is a cofactor required in the remethylation of homocysteine, too [5]. Homocysteine is converted to cysteine by vitamin B_6_. Deficiency of folic acid and vitamin B_6_ can also cause hyperhomocysteinemia. Hyperhomocysteinemia is known to increase oxidative stress and cause various diseases, including ischemic heart disease, ischemic stroke [6], Alzheimer’s disease [7], and Parkinson’s disease.

Vitamin B_12_ [8,9], folate [10], and vitamin B_1_ deficiencies [11] have been reported to induce brain atrophy. If so, cognitive decline may not be improved due to brain atrophy. Other factors including hyperhomocysteinemia [12,13], diabetes mellitus [14], and renal failure [15] can be the cause of brain atrophy. However, it was not clear which factors, especially which vitamin deficiencies, were associated with brain atrophy.

It is widely known that the extent of brain atrophy detected by MRI and cognitive functions are significantly correlated [16]. VBM (voxel-based morphometry) analysis is an objective index to evaluate the extent of brain atrophy based on 3D brain images; furthermore, voxel-based specific regional analysis system for Alzheimer’s disease (VSRAD) analysis with a region of interest (ROI) for AD set in the VBM, is widely used in Japan. The extent of the hippocampal atrophy can be quantified by the Z-score [4,5,17,18].

In this study, to clarify which vitamin deficiencies are associated with brain atrophy and to what extent, we aimed to investigate the relationship between hippocampal atrophy and mainly vitamin B_12_, folate, and vitamin B_1_ levels, using VSRAD analysis.

## 2 Methods

### 2.1 Study population

**Fig. 1:**
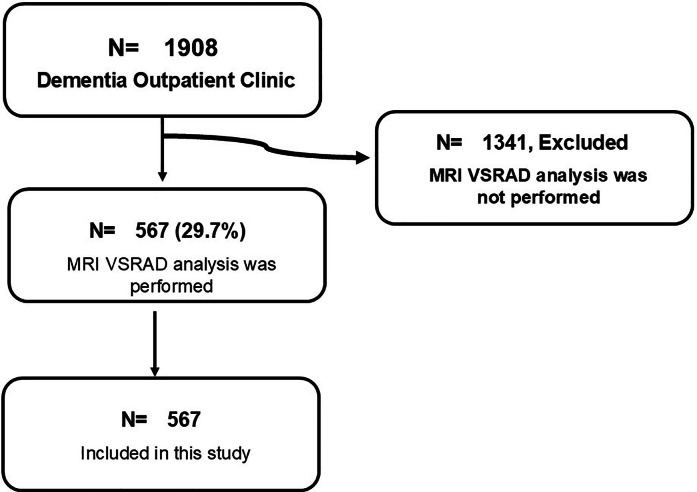
Flowchart showing how study participants are selected. Of the 1908 patients visiting Dementia Outpatient Clinic, MRI-VSRAD analysis was performed on 576 patients. A total of 567 patients were enrolled in the study.

Patients who visited the outpatient clinic for dementia at the University of Fukui Hospital, Nakamura Hospital, and Kimura Hospital between January 6 2008 and December 20 2022 were included in the study. Of the 1908 patients, 567 patients who underwent MRI-VSRAD were included in the analysis (Fig. 1). Patients with advanced dementia who did not receive adequate nutrition were not included in this study.

Vitamin B_12_, folate, vitamin B_1_, homocysteine, HbA1c, creatinine levels were estimated. Vitamin B_12_ levels were evaluated in 484 patients, folate in 494 patients, vitamin B_1_ in 440 patients, homocysteine in 483 patients, HbA1c in 555 patients, creatinine in 434 patients, MMSE of 561 patients, and education in 182 patients.

Cutoff values for vitamin B_12_ deficiency were < 233 pg/mL, folate deficiency was < 3.6 ng/mL, vitamin B_1_ deficiency was < 2.0 μg/dL, high homocysteine was 13.5 > μmol/L, high HbA1c was > 6.1 % NGSP, and high creatinine was > 0.79 mg/dL. Normal values at our hospital were adopted as cut-off value.

The participants were stratified based on the Z-score (hippocampal atrophy) into normal (Z-score < 2; mean age, 78.3 ± 8.6 years, *n* = 323) and hippocampal atrophy groups (Z-score ≥ 2; mean age, 79.9 ± 8.3 years; *n* = 244).

The association of vitamin B_12_, folate, vitamin B_1_, homocysteine, HbA1c, and creatinine levels with hippocampal atrophy were assessed. Additionally, age, MMSE (Mini Mental State Examination) and hippocampal atrophy were examined.

The Institutional Review Board of the University of Fukui approved this study (20180092).

### 2.2 Blood tests

An ADVIA Centaur XP Immunoassay System (Siemens Healthcare Diagnostics Manufacturing Ltd., Dublin, Ireland) and support equipment (Siemens Healthcare Diagnostics Inc. Diagnostics Inc., New York, NY, USA) were applied to measure vitamin B_12_, folate, and vitamin B_1_, levels on the same day. Creatinine was measured by enzymatic method by using Cygnus Auto CRE (Shino-Test Corporation, Tokyo, Japan). HbA1c levels were quantified by using High-Performance Liquid Chromatography (HPLC) (ADAMS™ A1c HA-8180 V, ARKRAY, Kyoto, Japan). Total homocysteine levels were measured using an atmospheric pressure ionization (API) 3200 LC-MS/MS system (SCIEX, Tokyo, Japan) [4,5].

### 2.3 Grading brain atrophy by VSRAD Z-score

All MRI scan at the University of Fukui Hospital and Nakamura Hospital were executed as previously described [4,5]. The VSRAD uses VBM to evaluate regional brain volumes by statistical comparison using brain imaging database of 80 healthy participants (54–86 years old) using Statistical Parametric Mapping (SPM). After standardize anatomically, isotropic 8-mm cubic smoothing was achieved to decrease individual variations in functional brain localization and approximate normal distribution. The Z-score was the main index, indicating the number of standard deviations of gray matter and white matter volumes as compared to that of the non-demented, normal participants.

In VSRAD advance, the target area was set in the medial temporal region, including the hippocampus and parahippocampal gyrus. Z-scores of 0–1 indicated little or no atrophy, 1–2 indicated some atrophy, 2–3 indicated considerable atrophy, and ≥ 3 indicated severe atrophy [4,5,17,18]

### 2.4 Statistical analysis

We presented the data as mean ± SD. We analyzed the differences between the two groups by means of the Mann-Whitney *U test*. Correlations were also evaluated using the Spearman's rank correlation coefficient when the data deviated from a normal distribution. Logistic regression analysis was performed to identify major factors associated with hippocampal atrophy.

Statistical analyses were done by IBM SPSS Statistics for Windows version 27(IBM Corp., Armonk, NY, USA).A *p*-value of < 0.05 was considered significant [19].

## 3 Results

### 3.1 Participant features

The mean age of the participants was 78.9 ± 8.5 years with 40 % (*n* = 227) male participants. Overall, 43 % of the total participants were in the hippocampal atrophy group. The mean Z-score, Z-score of the normal group, and that of the hippocampal atrophy were 2.3 ± 2.4, 1.1 ± 0.5, and 3.8 ± 3.1, respectively. The mean values of each parameter in the normal and hippocampal atrophy groups are presented in Table 2.

### 3.2 Biochemical parameters in hippocampal atrophy and normal groups

Vitamin B_12_ deficiency was detected in 7.9 % of the participants, folate deficiency in 11.4 %, vitamin B_1_ deficiency in 2.3 %, high homocysteine in 35.2 %, high HbA1c in 13.3 %, and high creatinine in 48.1 %. The mean MMSE score was 21.8 ± 5.1 points with median education (Median (IQR)) of 12(6–16) (Table 1).

**Table 1:** Patient characteristics (*N* = 567).

	Total (*N* = 567)
Age	78.9 ± 8.5
Male sex, %	40
MMSE	21.8 ± 5.1
Z-score	2.3 ± 2.4
Vitamin B12, pg/mL	547.9 ± 377.5
Folate, ng/mL	8.5 ± 18.7
Vitamin B1, μg/dL	3.9 ± 1.6
Homocysteine, μmol/L	14.0 ± 11.4
HbA1c, %	5.7 ± 0.1
Creatinine, mg/dL	0.89 ± 0.57
Education (Year) (Median(IQR))	12 (6–16)

Data are presented as mean ± SD.

MMSE, Mini Mental State Examination.

Furthermore, 4.6 % of the participants in the normal group and 12.2 % in the hippocampal atrophy group had vitamin B_12_ deficiency; 12.0 % of the participants in the normal group and 11.0 % in the hippocampal atrophy group had folate deficiency; 2.5 % of the participants in the normal group and 2.0 % in the hippocampal atrophy group had vitamin B_1_ deficiency. High HbA1c was observed in 13.6 % of the participants in the normal group and 12.6 % of the participants in the hippocampal atrophy groups. High creatinine levels were observed in 51.5 % of participants in the normal group and 44.2 % of participants in the hippocampal atrophy group. MMSE was 22.8 ± 4.8 and 20.5 ± 5 % in the normal and hippocampal atrophy groups, respectively. The median education level (Median(IQR)) was 12(6–16) and 12(6–16) in the normal and hippocampal atrophy groups, respectively (Table 2).

**Table 2:** Biochemical parameters in the hippocampal atrophy group and normal groups detected by VSRAD z score (*N* = 567).

	No hippocampal atrophy group (*N* = 323)	Hippocampal atrophy group (*N* = 244)	*p*-value
Age (Mean ±SD)	78.3 ± 8.6	79.9 ± 8.3	0.004
Male sex,%	49.2 (126/257)	41.8 (102/244)	0.502
MMSE (Mean ±SD)	22.8 ± 4.8	20.5 ± 5.2	<0.0001
Z-score (Mean ±SD)	1.1 ± 0.51	3.8 ± 3.1	<0.0001
Vitamin B_12_ deficiency, %	4.6 (12/259)	12.2 (26/213)	0.002
Folate deficiency, %	12.0 (33/276)	11.0 (24/218)	0.744
Vitamin B_1_ deficiency, %	2.5 (6/238)	2.0 (4/202)	0.719
High homocysteine, %	33.1 (90/272)	38.0 (80/211)	0.323
High HbA1c, %	13.6 (43/316)	12.6 (30/239)	0.716
High creatinine, %	51.5 (122/237)	44.2 (87/197)	0.129
Education (Year) (Median) (IQR)	12 (6–16)	12 (6–16)	0.120

Data are presented as mean ± SD.

MMSE, Mini Mental State Examination.

Vitamin B_12_, folic acid, vitamin B_1_, homocysteine, HbA1c, and creatinine levels were compared between the two groups. Furthermore, age and MMSE scores were assessed between the two groups. In the hippocampal atrophy group, the prevalence of vitamin B_12_ deficiency was significantly higher (*p* < 0.002), the mean MMSE score was significantly lower (*p* < 0.0001), and age was significantly higher (*p* < 0.004) than those in the normal group (Table 2).

### 3.3 Correlation between hippocampal atrophy and various parameters

The correlation of vitamin B_12_, folate, vitamin B_1_, homocysteine, HbA1c, and creatinine levels with hippocampal atrophy was also assessed. The correlation between age and MMSE and hippocampal atrophy was also examined. Correlations between hippocampal atrophy and prevalence of vitamin B_12_ deficiency (*p* = 0.002), MMSE score (*p* < 0.001), and age (*p* = 0.004) were detected (Table 3). As all data did not follow a normal distribution, Spearman's rank correlation coefficient was used for analysis.

**Table 3:** Correlations between hippocampal atrophy detected by VSRAD z score and various parameters.

Parameters	Univariate analysis	
	r	*p*-value
Age	0.122	0.004
Sex	−0.28	0.503
MMSE	−0.185	<0.001
Vitamin B_12_	−0.144	0.002
Folate	0.015	0.744
Vitamin B_1_	0.047	0.269
Homocysteine	0.045	0.323
HbA1c	0.015	0.716
Creatinine	0.026	0.536
Education	−0.128	0.068

MMSE, Mini Mental State Examination.

### 3.4 Factors contributing to hippocampal atrophy

Multivariate analysis revealed that vitamin B_12_ levels (odds ratio, 3.46; 95 % CI, 1.59–7.53; *p* = 0.002) and low MMSE score (odds ratio, 2.24; 95 % CI, 1.50–3.37; *p* < 0.0001) were associated with enhanced chance of hippocampal atrophy (Table 4).

**Table 4:** The logistic regression analysis of hippocampal atrophy and different parameters.

Parameters	Odds ratio	*p*-value	95%CI
Age	0.99	0.454	0.97−1.01
MMSE	2.24	<0.0001	1.50−3.37
Vitamin B_12_ deficiency	3.46	0.002	1.59−7.53
Folate deficiency	0.77	0.431	0.40−1.47
Vitamin B_1_ deficiency	1.28	0.475	0.65−2.52
High homocysteine	1.02	0.930	0.65−1.60
High HbA1c	0.89	0.703	0.52−1.56
High creatinine	0.79	0.539	0.39−1.64

MMSE, Mini Mental State Examination.

### 3.5 Correlation between Z-score and vitamin B_12_ levels

**Fig. 2:**
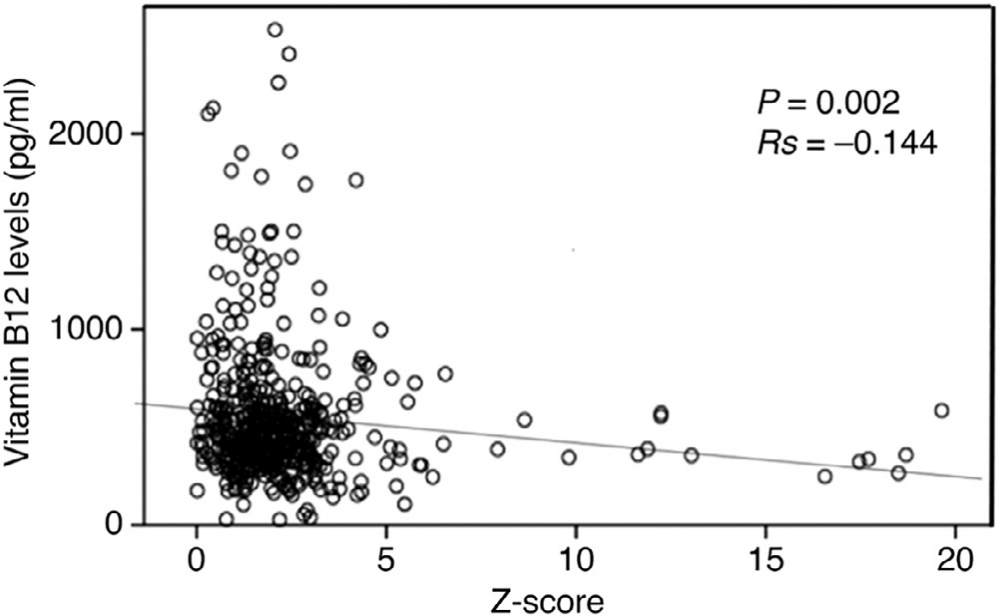
Correlation between z-scores and vitamin B_12_ levels MRI VSRAD Z-scores and plasma vitamin B_12_ values were inversely correlated.

Z-scores and vitamin B_12_ levels were correlated, and the corresponding correlation plot is presented in Fig. 2 (*p* = 0.002, *Rs* =−0.144).

### 3.6 Comparison between participants with and without vitamin B_12_ deficiency

Vitamin B_12_ deficiency contributed to hippocampal atrophy and comparisons of the participants with and without vitamin B_12_ deficiency revealed significant differences in the Z-score (*p* = 0.017), homocysteine (*p* < 0.001), folate (*p* = 0.004), and age (*p* = 0.018) (Table 5).

**Table 5:** Comparison between participants with and without vitamin B_12_ deficiency (*n* = 484).

	Normal vitamin B_12_ group (*N* = 446)	Low vitamin B_12_ group (*N* = 38)	*p*-value
Age (Mean ± SD)	78.1 ± 8.4	80.8 ± 8.9	0.018
Male sex, %	38.1 (170/446)	55.3 (21/38)	0.038
MMSE (Mean ± SD)	22.0 ± 5.0	21.3 ± 4.5	0.675
Z-score Mean ±SD	2.3 ± 2.6	2.5 ± 1.3	0.017
Vitamin B_12_ (Mean ± SD), pg/mL	580. 5 ± 375.3	166.8 ± 56.3	<0.001
Folate (Mean ±SD), ng/mL	8.1 ± 11.1	14.7 ± 56.0	0.004
Vitamin B_1_ (Mean ±SD), μg/dL	3.9 ± 1.6	3.5 ± 1.5	0.404
Homocysteine (Mean ±SD), μmol/L	12.8 ± 8.8	27.0 ± 24.1	<0.001
HbA1c (Mean ±SD), %	5.7 ± 0.9	5.5 ± 0.7	0.075
Creatinine (Mean ±SD), mg/dL	0.8 ± 0.7	1.5 ± 4.2	0.678
Education (Year) (Median) (IQR)	12 (6-16)	12 (6-12)	0.120

Data are presented as mean ± SD.

MMSE, Mini Mental State Examination.

## 4 Discussion

In the present study, vitamin B_12_ shortage was associated with hippocampal atrophy. Furthermore, correlations were observed between hippocampal atrophy and vitamin B_12_ value.

Vitamin B_12_ deficiency causes severe neurological complications in infants [20]. In 35 patients with vitamin B_12_ deficiency, aged 6 months to 2 years, thinning of the corpus callosum, elevation of the extra-axial space (28.6 %), brain atrophy (17 %), and diffuse symmetric white matter hyperintensities (5.7 %) have been reported [21]. Furthermore, severe cortical atrophy, cerebral hemorrhage, thinning of the corpus callosum, and delayed myelination have been reported on brain MRI in 21 infants aged 4 to 24 months with B_12_ deficiency [3]. In adults, there are some reports presenting the association of vitamin B_12_ deficiency and brain atrophy, too [20]. Kobe et al. reported that low vitamin B_12_ concentration within the normal range is poorer memory performance which is an effect that is partially mediated by hippocampal microsurgical integrity examined by MRI [22,23]. In patients with vitamin B_12_ deficiency and hippocampal atrophy, memory loss is associated with atrophy of the ammonis angle and dentate gyrus regions. In the present study, the MMSE scores were also associated with hippocampal atrophy. As widely believed, the hippocampus is associated with memory impairment [[24], [25], [26]].

In the present study, Vitamin B_12_ shortage was observed to be associated with hyperhomocysteinemia. Hyperhomocysteinemia has been reported to be associated with Vitamin B_12_ [4] and folate deficiencies [5,27]. Furthermore, hyperhomocysteinemia has been reported to be associated with brain atrophy [[28], [29], [30]], and vascular disorders [17]; however, reports on this association are controversial with some studies [26] reporting no association with hippocampal atrophy. In this study, no association was observed between hyperhomocysteinemia and hippocampal atrophy. Homocysteine has metabolic cofactors, including vitamin B_12_, folate, and vitamin B_6._ Homocysteine is also affected by various other factors including mutation in the 5,10-methylenetetrahydrofolate reductase (MTHFR) gene, renal failure, malignancy, immunological diseases, drugs, and lifestyle [31]. Therefore, in this study, characterizing brain atrophy based only on the relationship between vitamin B_12_ and homocysteine levels, was not easy.

In the previous studies, the association of brain atrophy and low vitamin B_12_ was reported. Vogiatzoglou et al. reported that low vitamin B_12_ was associated with increased risk of brain volume loss [32]. It was also reported that vitamin B_12_ deficiency can affect cognition by decreasing the total volume of the brain, and vitamin B_12_ status can affect the brain through multiple mechanisms at least in part [33]. VITACOG trial also implies that total homocysteine >13 μmol/L slow the cognitive decline by vitamin B_12_ supplementation [34]. Hooshmand et al. reported that high baseline levels of vitamin B_12_ and holotranscobalamin, which is the marker of functional vitamin B_12_, in the early phase were associated with a slower decline in brain volume [35].

Vitamin B_12_ deficiency causes treatable dementia and vitamin B_12_ supplementation has been reported to improve cognitive function [30,36]. The improvement in cognitive function may have been associated with improvement in mood disorders, at least in part [37]. VITACOG trial [30] found that hippocampal atrophy in mild cognitive impairment could be slowed by B vitamin treatment and that the effects on atrophy and on cognition were mainly due to administration of vitamin B_12_. However, cognitive impairment caused by brain atrophy may not improve with vitamin supplementation in advanced stage [38]. Thus, to prevent hippocampal atrophy caused by vitamin B_12_ deficiency, blood tests should be performed, and patients with loss of appetite, gastrointestinal tract surgery, and macrocytic erythroblastic anemia should be monitored for early detection and therapeutic intervention.

This study has some limitations. First, we were unable to exclude patients administered vitamin B_12_ prescription drugs or supplements. Patients who originally had low vitamin B_12_ levels and administered vitamin B_12_ supplementation would have had hippocampal atrophy, even if their vitamin B_12_ levels were normal at the time of entry. If we exclude patients who have supplemented with vitamin B_12_, a more significant difference may be obtained. Second, vitamin B_6_ levels were not estimated in this study, while considering hyperhomocysteinemia.

In conclusion, vitamin B_12_ deficiency is significantly associated with hippocampal atrophy. As previous studies including VITACOG study [30,39] have shown, cognitive decline and brain atrophy may be slower by vitamin B_12_ supplementation [40], necessitating earlier therapeutic interventions.

## Ethical approval and consent for participation

The protocol for this human clinical trial was approved by the University of Fukui Ethics Committee.

## Human rights

All materials were obtained in accordance with the standards set forth in the 1975 Declaration of Helsinki Principles as revised in 2008 (http://www.wma.net/en/10ethics/10helsinki/<http://www.wma.net/en/10ethics/10helsinki/>).

## Funding

This work was supported in part by a Grant-in-Aid for Scientific Research (No. JP 22K07392, 25K10679) from the Japan Society for the Promotion of Science, and a research grant from the University of Fukui (LSI20306).

## CRediT authorship contribution statement

**Asako Ueno:** Writing – review & editing, Visualization, Investigation, Data curation, Writing – original draft, Validation, Formal analysis, Conceptualization. **Tadanori Hamano:** Writing – review & editing, Visualization, Supervision, Resources, Methodology, Funding acquisition, Data curation, Writing – original draft, Validation, Software, Project administration, Investigation, Formal analysis, Conceptualization. **Miwako Nagata:** Supervision, Project administration, Resources, Investigation. **Tomohisa Yamaguchi:** Investigation. **Yoshinori Endo:** Investigation. **Soichi Enomoto:** Investigation. **Hirohiko Kimura:** Software, Methodology, Resources. **Masamichi Ikawa:** Investigation. **Osamu Yamamura:** Investigation. **Daiki Yamanaka:** Investigation. **Yohei Kimura:** Resources, Supervision. **Yasunari Nakamoto:** Funding acquisition, Supervision. **Yasuhiro Nishiyama:** Writing – review & editing.

## Declaration of competing interest

The authors declare the following financial interests/personal relationships which may be considered as potential competing interests:

Tadanori Hamano reports financial support was provided by Japan Society for the Promotion of Science. Tadanori Hamano reports financial support was provided by University of Fukui. If there are other authors, they declare that they have no known competing financial interests or personal relationships that could have appeared to influence the work reported in this paper.
